# New emerging targets in cancer immunotherapy: the role of TIM3

**DOI:** 10.1136/esmoopen-2019-000497

**Published:** 2019-06-12

**Authors:** Alex Friedlaender, Alfredo Addeo, Giuseppe Banna

**Affiliations:** 1 Oncology, Hopitaux Universitaires de Geneve, Geneva, Switzerland; 2 Oncology, Ospedale Cannizzaro, Catania, Italy

**Keywords:** TIM3, immunotherapy

## Abstract

Currently, the programmed death-1/programmed death ligand-1 and the cytotoxic T-lymphocyte-associated protein 4 are the two commonly targeted immune-checkpoint inhibition pathways. These drugs have significantly improved the prognosis of many cancer types. While immune-checkpoint inhibitors have revolutionised the treatment of many cancer types, the majority of patients still progress. Several treatment strategies have been pursued to improve current results. One approach is to combine two checkpoint inhibitors, currently with promising results in melanoma, renal cell carcinoma and a subset of non-small-cell lung cancer patients. The identification of new checkpoint targets could allow the field of immuno-oncology to evolve further. We will discuss one of the most promising immune-checkpoint targets currently under investigation, the T-cell immunoglobulin and mucin domain-3.

## Introduction

The immune system is the gatekeeper of cancer. Under normal circumstances, to counter tumour growth, effector T cells and tumour-infiltrating lymphocytes (TILs) represent the immune response to neoantigens and the process of immune surveillance.^1^ This process can be hampered by cancer through a dynamic process called immunoediting. This works by taking advantage of the mechanisms through which the immune system limits T-cell activation to regulate responses against self-proteins, and is mediated by the expression of immune-checkpoint proteins on T cells.^2^ Different treatment approaches may circumvent their effect and, just in recent years, the field of cancer immunotherapy has been revolutionised by immune-checkpoint inhibition.

Currently, the programmed death-1 (PD-1)/programmed death ligand-1 (PD-L1) and the cytotoxic T-lymphocyte-associated protein 4 (CTLA-4) are the two commonly targeted immune-checkpoint inhibition pathways. Essentially, blocking CTLA-4 removes inhibitory signals, allowing T cell activation and an immune response to a tumour antigen. Likewise, blocking the interaction between PD-1 and PD-L1 reinvigorates the antitumour T cell response.^2^ These drugs have significantly improved the prognosis of many cancer types. For example, 5-year survival for advanced non-small-cell lung cancer (NSCLC) has increased from 5% to 16%–25%,^3–5^ and for melanoma, from 22% to 34%–41%.^6^ While immune-checkpoint inhibitors (ICI) have revolutionised the treatment of many cancer types, the majority of patients still progress.^7^

Several treatment strategies have been pursued to improve current results. One approach is to combine two ICI, currently with promising results in melanoma, renal cell carcinoma and, perhaps, a subset of NSCLC patients.^8–10^ Adding ICI to chemotherapy is another combination, that is, gaining momentum, with significant benefit in small-cell lung cancer and NSCLC, triple-negative breast cancer and many trials ongoing.^11–15^ More recently, adding ICI to targeted therapy has shown improved survival in renal cell carcinoma.^16^ All of these strategies have shown the potential of combining treatments. The identification of new checkpoint targets could allow ICI to evolve further. We will discuss one of the most promising immune-checkpoint targets currently under investigation, the T-cell immunoglobulin and mucin domain-3 (TIM3).

## T-cell immunoglobulin and mucin domain-3

TIM3 is part of the TIM gene family which codes for proteins comprising TIM-1, TIM-3 and TIM-4. They are type-I cell-surface glycoproteins composed of signal peptides, extracellular Ig V domains, mucin-like and transmembrane domains and have an intracellular cytoplasmic tail.^17^

TIM3 can be expressed on both tumour and immune cells. It is expressed on multiple immune cells, including type 1 T helper (Th1) cells, Th17 and CD8+ T cells, TILs, regulatory T cells (Tregs) and innate immune cells. Th1 cells participate in antitumour cell-mediated immunity, as do Th17, via proinflammatory cytokines. On the other hand, CD8+ are cytotoxic T lymphocytes that bind their target antigen to destroy potential threats. TIM3 contributes to immune tolerance. During chronic infections, TIM3 is upregulated in CD8+ cells and in the presence of cancer, specifically in CD8+ TILs.^18^ TIM3 overexpression in CD4+ T cells can also be a sign of more aggressive or advanced disease. Similarly, it is often overexpressed on cancer cells and associated with an aggressive clinical course and poor survival.^19^

Unlike other currently known immune checkpoints, after T-cell activation, the upregulation of TIM3 is specific only to IFN-γ producing T-cells: CD4+ and CD8+ cells.

**Figure 1:**
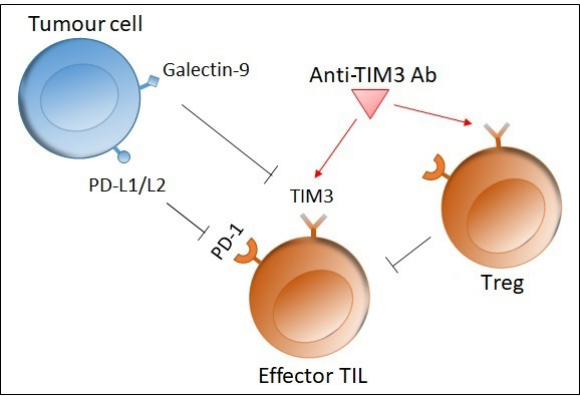
Role of Tim3. The interaction between Tim3 on an effector T cell and galectin-9 on a tumour cell inhibits the immune response by inducing apoptosis in the T cell. Tim3 is also upregulated on Treg, which in turn inhibit effector T cell function. Blocking Tim3 with a mAb thus enhances T cell proliferation and immune function. L1/L2, ligand 1/2; mAb, monoclonal antibody; PD, programmed death; TIL, tumour-infiltrating lymphocyte; Tim3, T-cell immunoglobulin and mucin domain-3; Treg, regulatory T cell.

Once TIM3 binds to its ligand, galectin-9, it inhibits the cell-mediated immune response by triggering cell death in TIM3-expressing T cells.^20^ As such, TIM3 overexpressing CD4+ and CD8+ T cells respond less to antigenic stimulation. TIM3 plays a role in T-cell exhaustion during chronic immune stimulation, such as in cancer. This phenomenon is characterised by the loss of T-cell effector functions, expression of multiple inhibitory receptors and an altered transcriptional programme.^19^ It has also been observed that in TILs, TIM3 is overexpressed in CD4+ and CD8+ T cells, while, in the same population, peripheral T-cells show minimal TIM3 expression.^19^ Moreover, blocking the TIM3 pathway can enhance tumour antigen-specific T cell proliferation and their ability to produce proinflammatory cytokines.^19 21^ Finally, TIM3 blockade inhibits Treg function and myeloid-derived suppressor cell function, contributing to an improved immune response.^19^ Thus, TIM3 can regulate both innate and adaptive immune responses (figure 1).

As PD-1 overexpression is another marker of T cell exhaustion, the dual blockade of these two pathways (PD-1/PD-L1 and TIM3) may be synergistic and more effective in restoring the T-cell proliferation and cytokine production.^18^ It is interesting to note that in early tumour development, most TILs do not express PD-1 and TIM3, while in more advanced stages, both proteins are frequently expressed.^22^ In preclinical mouse studies of solid tumours, CD8+ TILs coexpressing TIM3 and PD-1 exhibit profound defects in T cell effector function. Targeting PD-1 or TIM3 allows the restoration of immune function in these cells, but the greatest impact on controlling tumour growth appears when both targets are treated simultaneously.^18^ In another murine model, this time of NSCLC, TIM3 was upregulated in the case of progression after an initial response to anti-PD-1 ICI. The addition of a TIM3 monoclonal antibody (mAb) conveyed a survival advantage. Thus, in addition to being a marker of immune exhaustion, TIM3 could be a targetable biomarker associated with adaptive resistance to PD-1 inhibition.^23^

### Anti-TIM3 agents under development

The inhibition of TIM3 by mAbs is being investigated as single blockade, with combination strategies or with bispecific mAbs. From a biological point a view, it is a very promising target, justifying the 10 ongoing phase I trials (table 1).

**Table 1:** Tim3 inhibition

Name of compound	Mechanism of action	Study phase	Company	Trial ID*	Tumour type	Target accrual
*Single TIM3 blockade*						
Sym023	Anti-TIM3 mAb	I	Symphogen A/S	NCT03489343	Solid tumours and lymphoma	48
INCAGN02390	Anti-TIM3 Ab	I	Incyte	NCT03652077	Solid tumours	76
*Combination TIM3 blockade*						
LY3321367 ± LY3300054	Anti-TIM3 ± anti-PD-L1 mAb	I	Eli Lilly	NCT03099109	Solid tumours	196
Sym021 ± Sym023	Anti-PD1 ± anti-TIM3	I	Symphogen	NCT03311412 (arm B)	Solid tumours, lymphoma	102
MBG453 ± PDR001	Anti-TIM3 ± anti-PD1 mAb	I/II	Novartis	NCT02608268	Solid tumours	250
BGB-A425 + Tislelizumab	Anti-TIM3 + anti-PD1 mAb	I/II	BeiGene	NCT03744468	Solid tumours	162
TSR-022 ± TSR-042	Anti-TIM3 ± anti-PD1 mAb	I	Tesaro	NCT02817633	Solid tumours	627
TSR-022 + TSR-042 + chemo	Anti-TIM3 + anti-PD1 + chemo	I	Tesaro	NCT03307785 (parts F, H, I)	Solid tumours	168
*Bispecific TIM3 blockade*						
RO7121661	Anti-PD-1/TIM3 bispecific Ab	I	Hoffmann-La Roche	NCT03708328	Metastatic melanoma and NSCLC	280
LY3415244	Anti-PD-L1/TIM3 bispecific Ab	I	Eli-Lilly	NCT03752177	Solid tumours	117

*Clinicaltrial.gov ID.

Ab, antibody;mAb, monoclonal antibody;TIM3, T-cell immunoglobulin and mucin domain-3;chemo, chemotherapy

Combination therapies include immuno-oncology (IO)–chemotherapy and IO–IO approaches. IO–chemotherapy is a rapidly growing field based on the hypothesis that cytotoxic chemotherapy may enhance the tumour immunogenicity.^11–13^ While it increases response rates and survival, there is room for improvement. The combination of PD-1 and CTLA-4 mAbs has earned its role in advanced renal cancer and melanoma^24^ and has been shown to increase progression-free survival in a subset of NSCLC patients,^8^ although it is associated with a significant increase in immune-related side effects. The biological rationale of combined immune regulatory pathway inhibition is to reverse T cell exhaustion and achieve an additive or synergistic effect and eventually a stronger immune response towards cancer antigens. Further IO–IO combinations are under development, among which the addition of a TIM3 inhibitor to a PD-1 inhibitor. TIM3 is upregulated on PD-1 antibody (Ab)-bound TILs in mice with lung cancers that initially responded to PD-1 blockade, then progressed. The addition of an anti-TIM3 Ab conveyed a clinical and radiological advantage, doubling survival time. Biologically, it led to an increase in IFN-γ production and T cell proliferation, thus reversing cell exhaustion.^23^ In animal models, a synergistic effect of these checkpoint inhibitors in reducing tumour cells has been reported.^18^ One ongoing trial combines dual checkpoint inhibition with chemotherapy, aiming to reap the benefits of both therapeutic strategies (table 1).

With a similar rationale, a different approach entails the creation of bispecific Abs that can simultaneously target two oncogenic antigens or epitopes, a strategy with promising efficacy and safety preliminary results. Like single-target Abs, they can enhance antitumour effects of the immune system by eliciting the non-major histocompatibility complex-dependent effector T-cell activity.^25^ They include two major categories. The first comprises those with a Fc region, with an IgG-like format, with Fc-mediated effector functions. It relies on cytotoxic mechanisms resulting from the interaction between the Fc portion of the Ab and the Fc receptors or complement factor C1q. This is achieved through Fc-mediated effector functions including antibody-dependent cell-mediated cytotoxicity (ADCC) and antibody-dependent cell-mediated phagocytosis (ADCP), and complement-dependent cytotoxicity. As a reminder, ADCC involves an effector cell targeting a tumour cell bound to an antibody. ADCP is a process by which phagocytic effector cells such as macrophages internalise and degrade target cells marked by an Ab. In complement-dependent cytotoxicity, the IgG-like antibody binds the tumour antigen, forming a membrane attack complex, resulting in tumour lysis.

The second category of bispecific Abs lacks a Fc region and relies exclusively on the binding of the Ab to the cancer antigen to exert its effect, altering biological responses and possibly triggering apoptosis.^26 27^ Biologically, these bispecific Abs can be further divided into three categories: cytotoxic effector T-cell redirectors, tumour-targeted immunomodulators and dual immunomodulators. The former engages T-associated antigens and the T-cell receptor/CD3 complex, directing the T-cell cytotoxicity at tumour cells. However, CD3 can recruit T-cells indiscriminately, thus increasing the risk of immune-related adverse events.^28^ Tumour-targeted immunomodulators bind a tumour-associated antigen and an immunomodulating receptor and are activated by the binding of the antigen, thus allowing for a tumour-specific T-cell response with lower risk of immune-related adverse events. Finally, dual immunomodulators target two immunomodulating proteins resulting in the blockade of inhibitory checkpoint pathways, counteracting tumours’ immunosuppressive mechanisms.^28^ The latter category comprises the two PD-1/TIM3 bispecific Abs currently under investigation in phase I trials (table 1). The possible advantages of bispecific Abs are a lower toxicity and a decreased risk of escape signalling, by simultaneously binding and inhibiting both targeted pathways.

The only data currently available are a preliminary analysis from the phase I Amber trial. It includes an anti-TIM3 Ab, TSR-022, alone and in combination with an anti-PD-1 Ab. At the time of analysis, data were presented for 39 NSCLC patients who had progressed following initial anti-PD-1 treatment. They were given TSR-042 at a fixed dose of 500 mg and TSR-022 at either 100 mg (14 patients) or 300 mg (25 patients), every 3 weeks. Among 11 evaluable patients at the 100 mg dose, 1 had partial response (PR), 3 stable disease (SD), while in the 300 mg cohort, among 20 evaluable patients, 3 had PR and 8 SD. All responses were in PD-L1-positive patients. Analysing only the 12 PD-L1-positive patients, 4 had PR and 6 SD. Further analysis is underway with a 900 mg dose expansion cohort, as pharmacokinetic analysis showed that 300 mg are not enough to maintain the maximal pharmacodynamics effect. The current dosage appears to have a well-tolerated safety profile. Thus, in spite of low response rates, disease control rates are promising, at 55% overall and 83% in the PD-L1-positive subgroup.^29^

## Conclusions

TIM3 inhibition represents an intriguing target on a pathophysiological level, as a single target or in combination with PD-1 blockade. As stated, the preclinical data support the use of combined TIM3 and PD-1 blockade, as the phenotype of exhausted T cells in cancer expresses both markers and there appears to be a synergistic effect in immune restoration.^18 23^ Based on these data, we believe the potential role of TIM3 inhibition is most likely with concomitant PD-1 blockade, as initial treatment or as salvage therapy after anti-PD-1 failure. The preliminary results from the Amber trial suggest that PD-L1 expression (>1%) may be a predictive biomarker for combined blockade, but this requires confirmation.^29^

Data currently available are insufficient to hypothesise about the future of this target and results from the listed trials (table 1) are eagerly awaited. If these treatments prove to be successful with a good safety profile, TIM3 inhibition could further shape the landscape of lung cancer treatment.
